# Impact of anxiety-related conditions on nursing students’ academic excellence

**DOI:** 10.4102/curationis.v47i1.2571

**Published:** 2024-10-04

**Authors:** Hlengiwe D. Seshabela, Simangele Shakwane

**Affiliations:** 1 Department of Health Studies, College of Human Sciences, University of South Africa, Pretoria, South Africa; 2 Department of Health, Gauteng College of Nursing, Pretoria, South Africa

**Keywords:** academic excellence, anxiety-related conditions, counsellors, impact, nursing education institution nurses, nursing students

## Abstract

**Background:**

Nursing students often experience high anxiety and depression because of the demanding nursing curriculum. This mental strain can harm their academic performance. As a result, nurse educators need to assess how anxiety impacts nursing students’ academic achievements.

**Objectives:**

To evaluate and describe the knowledge of counsellors and nurse educators regarding the impact of anxiety-related conditions on nursing students’ academic excellence in selected nursing education institutions.

**Method:**

A qualitative descriptive phenomenological design was used to evaluate and describe the perceptions of counsellors and nurse educators about anxiety-related conditions on nursing students’ academic excellence. Participants were purposively selected, and semi-structured interviews were utilised to collect data. Four counsellors involved in psychosocial support of nursing students who participated in individual semi-structured interviews, and 20 nurse educators participated in focus group interviews, with 5 participants per group. Data were recorded and transcribed. Transcripts were analysed using Giorgi’s (2009) four data analysis steps.

**Results:**

Four themes emerged from data analysis: understanding of anxiety-related conditions, responses to anxiety-related conditions, influences of anxiety-related conditions and support of nursing students with anxiety-related conditions. The findings highlighted the significance of recognising the effects of mental health issues on nursing students.

**Conclusion:**

The study revealed the factors influencing nursing students’ responses to and the support provided for anxiety-related conditions affecting their academic performance.

**Contribution:**

The importance of prioritising mental health support for nursing students is to ensure they complete their qualifications on time.

## Introduction

Nursing students are exposed to constant stressors that make them more vulnerable to mental health problems and affect their emotional and physical well-being (Gangadharan & Madani 2018:1). Fernandes et al. (2018:2) claim that nursing students exhibit higher levels of anxiety compared to other university students because of factors such as clinical practice, relationships with patients, fear of making mistakes and increased academic demands. The clinical learning environment is characterised by psychological, physical and organisational factors that impact nursing students’ learning because of the complicated nature of patient conditions and the complexity of the learning process (Najafi et al. 2018:2). Anxiety-related conditions pose a significant public health concern, leading to stigma, discrimination and neglect within the healthcare system, resulting in poor health outcomes, isolation and high suicidal rates (Meyer, Matlala & Chigome 2019:1). Anxiety is a feeling of unease and apprehension related to the anticipation of danger, the course of which is often unknown, and is accompanied by behaviour disturbances such as impairments in social and occupational functioning (Townsend & Morgan 2018:560). The National Institute of Mental Health (NIMH) (2022) describes anxiety as the normal part of life when worrying about everything, but if it continues over time, it becomes an anxiety disorder. Anxiety disorders are characterised by a wide range of cognitive and somatic disorders with an elevated prevalence of dysfunction, particularly depression (Ferber et al. 2021:1). Within nursing education institutions (NEIs), anxiety negatively affects academic success leading to mental blocks during assessments and hindering nursing student performance (Esmaeelzadeh et al. 2018:1). Anxiety contributes significantly to the risk of self-harm and suicide because of the way symptoms affect coping in the face of present psychosocial stressors (Foster et al. 2021:158). Anxiety and depression directly influence nursing student performance, leading to low self-esteem and insecurity, and they are also exposed to emotional overload related to patient safety (Coelho et al. 2020:2).

The prevalence of anxiety-related conditions contributes to the throughput rate in South Africa, accounting for 18% of cases compared to other mental health disorders (Wagner et al. 2022:1). Nursing students worldwide encounter high levels of stress because of the demanding curriculum, which often lead to anxiety and depression (Hwang & Kim 2022:1). Challenges faced by nursing students include financial constraints, household responsibilities and being challenged as a student and having difficulty balancing work and academic life (Jacobs, Scrooby & Du Preez 2019:6). Maintaining academic achievement and success while retaining nursing students in NEIs is an ongoing global challenge that requires timely identification and intervention of nursing students at risk because of anxiety (Mthimunye & Daniels 2019:2). The presence of anxiety, its manifestations and its level of intensity level represent an essential aspect of any mental health assessment and intervention (Middleton 2020:323). Inadequate integrated healthcare services contribute to increased levels of anxiety among nursing students, which is often a result of suboptimal clinical practice (Esmaeelzadeh et al. 2018:1). Fadana and Vember (2021:1) emphasise that insufficient work-integrated learning leads to a decline in practice quality.

In African countries, the study conducted among health science students revealed an alarming prevalence of anxiety and depression: 88.8% suffered from depression while 82.6% suffered anxiety because of workload and examination pressures (Agyapong-Opoku et al. 2023:2). Furthermore, the influence of these health problems on the academic performance of health professionals was evident, as students experience depression and anxiety exhibited decreased psychomotor concentration and tended to avoid learning activities that negatively affect their academic performance (Baluwa et al. 2021:2).

In South Africa, a high failure rate among nursing students leads to demotivation and attrition (Langtree, Razak & Haffejee 2018:90). To address this issue, South African NEIs have established counselling departments to promote to promote Positive Practice Environments (PPEs) consistent with the Strategic Plan for Nurse Education, Training and Practice (2012/2013–2016/2017). These departments aimed to support nursing students and alleviate their challenges within NEIs. However, despite support, anxiety-related conditions persist among nursing students. As postulated by Savitsky, Findling and Hendel (2020:1), anxiety remains an integral part of nursing students’ experiences because of factors such as heavy workloads, examinations, complex interpersonal dynamics and the challenges presented by the clinical environment, including caring for terminally ill patients. Therefore, prioritising the psychosocial needs of nursing students and improving their mental well-being are crucial to create a capable and resilient generation for the future (Fattahi et al. 2020:6).

### Problem statement

Nursing success is desired but is challenged by high levels of anxiety, which negatively impacts academic performance and increases attrition rates (Mthimunye & Daniels 2020:200). Nursing students come from diverse backgrounds in race, gender, religion, culture and socioeconomic class, which require recognition and embrace, impacting retention and academic success (Green 2020:1). Diversifying the workforce is important for improving healthcare delivery, particularly by addressing mental health issues among nursing students during their training, thereby improving academic success and retention (Zerwic et al. 2018:1). Nursing education is consistently associated with anxiety among nursing students because of heavy course loads, complex interpersonal relationships, examinations and caring for terminally ill patients (Savitsky et al. 2020:1). The researcher recognised the need to assess the knowledge of both counsellors and nurse educators about anxiety-related conditions among nursing students, resulting from poor academic performance and high levels of anxiety levels.

### Objective

The objective of the study was to evaluate and describe the knowledge of counsellors and nurse educators regarding the impact of anxiety-related conditions on nursing students’ academic excellence in selected NEIs.

## Research methods and design

Descriptive phenomenology was conducted to evaluate and describe the knowledge of counsellors’ and nurse educators’ impact of anxiety-related conditions on nursing students and academic excellence at selected NEIs. In its pursuit, descriptive phenomenology aims to discover and describe the core of participants’ meaning of their experiences, while the researcher brackets her own experiences during data collection and data analysis (Kim et al. 2020:17; Shorey & Ng 2022:2). The design utilised is a type of research that explores and provides deeper insights into real-world problems, by asking open-ended questions such as ‘how’ and ‘why’ (Tenny, Brennan & Brannan 2022:1). Data analysis followed Giorgi’s (2009) four-step method, comprehensively describing how these professionals perceived anxiety-related conditions. Verbal extracts from counsellors and nurse educators supported the related data.

### Setting

The study was conducted in three selected South African NEIs in Gauteng province in the City of Tshwane. These three NEIs were selected for feasibility, availability and accessibility of participants relevant to the study. The selected NEIs were accredited by the South African Nursing Council (SANC) to provide R. 425 legacy programme, a diploma in nursing (general, community and psychiatry) and midwifery, which is in teaching until 2024, and the two new nursing education programmes: R.171 (3-year diploma) and R.174 (4-year bachelor’s degree).

### Population and sampling

Two groups were recruited to participate in the study: nurse educators who taught undergraduate education and training programmes and had 3 years and above of teaching experience, and counsellors who worked in the counselling department for over 2 years in selected Gauteng Province NEIs. Participants who met the inclusion criteria and were willing to participate were purposively sampled based on their experiences with nursing students. Purposive sampling is a method in which the researcher purposefully selects participants based on their knowledge of the phenomena under study (Polit & Beck 2021:799). Four counsellors and 20 nurse educators with various experiences of anxiety participated in the study.

### Data collection

The semi-structured nature of the interviews allows for a flow of conversation that focusses on the purpose of the study, which is particularly useful for exploratory, probing and summarising purposes (Brink, Van der Walt & Van Rensburg 2018:143). All interviews were conducted virtually as the country was under Alert Level 3 because of coronavirus disease 2019 (COVID-19) pandemic restrictions. An interview guide consisted of two sections: Section 1, which covered demographic data, and Section 2, which included open-ended semi-structured questions to evaluate knowledge related to the anxiety of counsellors and nurses’ educators. All participants were asked the same questions, and probing was used for clarity; for example, the researcher asked for clarity and probing for more information, ‘What do you mean?’ or ‘Please explain’. Semi-structured telephone interviews were conducted to obtain information from counsellors who consented to participate in the study. The duration of the interviews was between 30 and 45 min and varied depending on the degree of knowledge of anxiety among nursing students. In qualitative research, data saturation is important, and it requires the researcher to collect data to the point that no further data can be collected (Mwita 2022:2). Despite reaching data saturation at Participant 3, the researcher interviewed another counsellor to confirm that no new information emerged.

Twenty nurse educators were recruited for focus group interviews via individual contacts, study introduction and willingness assessment. The researcher informed participants that interviews would be focus groups conducted through Microsoft Teams. Relevant documentation was emailed to willing participants, followed by a phone call for any questions. Following receipt of the demographic data forms, participants signed an informed consent form and a confidentiality agreement to respect the privacy of fellow participants. The researcher scheduled a meeting for a focus group; a link was sent to each participant individually before the meeting, ensuring that their identities remained confidential. Participants were asked for permission to audio-record the discussion.

Participants were asked to raise their hands when they needed to speak, and each participant was given a number for coding. The focus group interviews took 1 h and 30 min. All interviews were audio-recorded. The researcher conducted four focus group interviews with five nurses and educators in each group. In the third group, no new data were obtained from the participants, but the researcher continued with additional groups to ensure that the data received were well understood. Data were collected from three selected NEIs from May 2021 to September 2021. Table 1 presents the interview guide used during the discussions of participants, counsellors and nurse educators.

**TABLE 1:** Interview guide.

1.	What is your understanding of anxiety-related conditions?
2.	Please share your experiences with me of nursing students who have anxiety-related conditions.
3.	Share your understanding of the factors that may increase anxiety-related conditions in your case.
4.	What mechanisms are currently used to support you in coping when you experience anxiety-related conditions?
5.	What mechanisms do you use to cope with the anxiety-related conditions experienced?
6.	To what extent are the mechanisms currently used effective?

### Data analysis

The researcher maintained a close connection with the narratives shared by the participants and aimed to provide an authentic account consistent with the researcher’s interpretations of the participants. This process resulted in the creation of a comprehensive description of the study’s results. Data were phenomenologically analysed using Giorgi’s (2009) four-step data analysis.

In Step 1, the researcher reviewed the transcriptions thoroughly and included multiple readings of the transcribed interviews to fully understand the entire data set. Step 2 focussed on identifying units of meaning through a careful discrimination process. Step 3 involved transforming participants’ natural expressions into expressions aligned with phenomenological psychology. This step entailed the collection of meaning units and the construction of a meaningful structure. Step 4 summarised participants’ experiences and articulated the core meaning of their knowledge. Themes and sub-themes emerged and were documented. The researcher and independent co-coder agreed with the findings of the semi-structured interviews.

The immersion process was characterised by repeated readings of the transcripts, allowing the researchers to become familiar with the content, including the participants’ voices and unique perspectives, emotions and meanings attached to their words. The meaning of data was determined by synthesising the understanding gained from the immersion process. The researchers aimed to discern patterns, themes and insights from the data.

### Ethical considerations

Ethical approval was obtained from the Research University of South Africa’s Health Research Ethics Committee (reference no.: 08039992_CREC_CHS_2021) and the Gauteng Department of Health (reference no.: 2021_03080). Permission to conduct the study was obtained from the head of each NEI. The participants who volunteered and were willing to participate were forwarded all the research documents. Participants were advised to use a password to maintain confidentiality when returning the informed consent and demographic data form. All interviews were recorded and transferred to a password-controlled file on the researcher’s computer. Verbatim transcriptions were written in Word documents and kept in a password-controlled file on the researcher’s computer. All the identifiable information was separated and set aside before sharing the data with an independent coder, and she signed a confidential agreement to protect the participants.

#### Trustworthiness

Trustworthiness was enhanced using Lincoln and Guba’s 1985 framework cited in Brink et al. (2018:157), which included credibility, dependability, confirmability, transferability and authenticity criteria. The researcher built credibility by fostering trust through prolonged telephonic engagement with participants, allowing for open and free discussions. During interviews, the participants were allowed to share their experiences without being rushed. Using a thick description approach, a thorough description of the data was employed to enable readers to assess the relevance of data in a similar context. Reliability was upheld by providing evidence in a manner that if the research process were replicated with comparable participants, would yield consistent results. Confirmability was enhanced by using the services of an independent coder who conducted data analyses independently. The researcher and co-coder independently discussed and agreed on the presented themes and sub-themes. For transferability, a rich and in-depth description of participants’ responses was presented under the findings. Authenticity was validated by evidence of audio recordings of data from semi-structured interviews and verbal transcripts. A dense data description with direct quotes from participants was used to support the findings.

## Results

### Participants’ characteristics

The participants were divided into two groups: counsellors and nurse educators.

#### Counsellors

The counsellors were four female nurses with psychiatric qualifications that allowed them to support nurses in selected NEIs. Their ages ranged from 46 to 62 years. Three had Master’s degree in nursing education, and one had an honours degree in psychology.

They all had no additional qualifications in counselling nursing students. Table 2 presents a summary of the counsellors’ characteristics.

**TABLE 2:** Participants’ characteristics: Counsellors.

Participant Code	NEI	Age (years)	Gender	Counselling experience (years)	Highest qualification	Additional qualifications
003-B-21-01	003	57	Female	3	Master’s degree in nursing education	None
003-B-21-02	003	62	Female	3	Master’s degree in nursing education	Certificate in psychology and marriage counselling
001-B-21-03	001	51	Female	4	Master’s degree in nursing education	None
002-B-21-04	002	46	Female	6	Honours degree in psychology	None

NEI, nursing education institution.

#### Nurse educators

Nurse educators were all registered nurses with the South African Council with nursing education qualifications that enabled them to teach and support nursing students in three selected NEIs. Twenty nurse educators participated in the study, of whom 19 were females and 1 male. Their ages ranged from 30 to 62 years. Five had Master’s degree, and 12 were studying for their Master’s degree. Table 3 shows the summary of the nurse educators’ characteristics.

**TABLE 3:** Participants’ characteristics: Nurse educators.

Participant code	NEI	Age (years)	Gender	Experience as a nurse educator (years)	Highest qualifications
001-C-21-FG01	001	35	Female	3	Master’s degree in public health
001-C-21-FG02	001	37	Female	4	BCur degree
001-C-21-FG03	001	54	Female	5	Master’s degree in nursing education
001-C-21-FG04	001	62	Female	8	BCur degree
001-C-21-FG05	001	58	Female	15	BCur degree
001-C-21-FG06	001	62	Female	25	BCur degree
001-C-21-FG07	001	30	Female	3	BCur degree
001-C-21-FG08	001	34	Male	3	BCur degree
001-C-21-FG09	001	47	Female	4	BCur degree
001-C-21-FG10	001	59	Male	8	BCur degree
001-C-21-FG11	001	56	Female	7	BCur degree
001-C-21-FG12	001	50	Female	3	BCur degree
001-C-21-FG13	001	49	Female	5	BCur degree
001-C-21-FG14	001	52	Female	6	Master’s degree in nursing education
001-C-21-FG15	001	60	Female	26	BCur degree
002-C-21-FG16	002	60	Female	10	Master’s degree in nursing education
002-C-21-FG17	002	59	Female	5	BCur degree
002-C-21-FG18	002	50	Female	4	Master’s degree in nursing education
002-C-21-FG19	002	59	Female	3	BCur degree
002-C-21-FG20	002	43	Female	3	Master’s degree in nursing education

NEI, nursing education institution.

### Semi-structured interview findings

The presentation of the counsellors and nurse educators’ findings are merged.

Four themes emerged during the data analysis, which were (1) understanding of anxiety-related conditions, (2) influences of anxiety-related conditions, (3) responses to anxiety-related stressors and (4) support of nursing students with anxiety-related conditions. Eleven sub-themes emerged from data analysis as shown in Table 4.

**TABLE 4:** Summary of themes and sub-themes.

Themes		Sub-themes
1.	Understanding of anxiety-related conditions among nursing students	1.1 Severe level of anxiety 1.2 Panic attack
2.	Influences of anxiety-related conditions	2.1 Academic-related pressures 2.2 Lack of a supportive environment 2.3 Chronic medical health challenges 2.4 Social influences
3.	Responses of anxiety-related conditions among nursing students	3.1 Physical symptoms 3.2 Emotional symptoms
4.	Support of nursing students with anxiety-related conditions	4.1 Practical advice given to nursing students with anxiety-related conditions 4.2 Referral to relevant professionals 4.3 Availability of support system

### Theme 1: Understanding of anxiety-related conditions among nursing students

Participants shared their understanding of the anxiety-related conditions among nursing students as severe feelings of fear, uncertainty and being overwhelmed. They explain that they [participants] need to understand anxiety-related conditions among nursing students, as they can better manage them more effectively with knowledge compared to those who do not. Two sub-themes emerged from this theme.

#### Sub-theme 1.1: Severe level of anxiety

The participants mentioned that nursing students might experience significant concern, being overwhelmed, edginess, loss of hope and uncertainty. This feeling interferes with daily activities. This was explained as follows:

‘The feeling of fear, anxiety and uncertainty interferes with nursing students’ daily activities. It can be caused by social or emotional that can push them so much that it affects academics as the level of anxiety is severe.’ (003-B-21-01)

‘Nursing students feel overwhelmed with anxiety, lose hope and self-confidence.’ (003-B-21-02)

Additionally, they also felt that nursing students lack resilience, as they spend a lot of time very worried and fear the unknown:

‘My understanding is that anxiety is fear, it might be imagined by somebody that something bad will happen, uncertainty feeling and lack resilience.’ (001-C-21-FG05)

‘My understanding of anxiety is a fear of the unknown, overwhelming feeling, their hands [*nursing students*] trembling, nervous, worrisome about what will happen, high level of anxiety.’ (002-C-21-FG17)

Whenever nursing students are anxious, they become emotionally disturbed in such a way that they cannot cope with other activities and become uneasy. Their academic performance declines because of their inability to cope. They lose confidence in their academic progress because of a lack of concentration, increasing the failure rate.

#### Sub-theme 1.2: Panic attacks

The knowledge of nursing students with anxiety-related conditions was expressed as a state of panic, hysteria and not knowing what to do next. This usually happens when they feel that they are not supported while practising nursing skills. Increased levels of anxiety and panic become evident when nursing students face tests and examinations. Also, when the objectives are unclear, they feel unprepared to participate in their academic journey. This was narrated as follows:

‘Some of the nursing students panic a lot requiring our continued support especially when objectives are not clear. Most of them [*nursing students*] need reassurance for them to be strong and continue with their studies.’ (001-B-21-03)

‘When nursing students are not prepared for their examinations, it is obvious that they will panic. Some even absent themselves when they are allocated to present in class.’ (001-C-21-FG03)

Based on the results, nursing students experience panic attacks because of unpreparedness for academic activities. Therefore, nurse educators must prepare nursing students well for their presentations, tests and exams, as anxiety worsens when underprepared.

### Theme 2: Influences of anxiety-related conditions among nursing students

Participants narrated anxiety influences among nursing students related to personal, academic and medical conditions and environmental pressures that increase anxiety-related conditions. Four sub-themes emerged from the participants’ data: academic-related pressure, lack of a supportive environment, chronic medical health and social issues.

#### Sub-theme 2.1: Academic-related pressures

According to participants, academic-related pressures are considered a secondary cause of anxiety-related conditions among nursing students. Primary pressure stems from the nursing students’ social backgrounds and pre-existing factors. Most nursing students report problems associated with social dysfunction, history of trauma and physical health issues when they start their academic journey. These issues overshadow academic pressure. According to the *South African Nursing Act guidelines* (South African Nursing Council 2005:2), nursing programmes are designed to emphasise the practical application of theory in an authentic, work-based context addressing specific competencies. This is expected to be done by nursing students in their work-integrated learning. This was expressed in the following experiences:

‘Most symptoms are observed pre-examination/test and after examinations and test when the students have failed due to mental block.’ (002-B-21-04)

‘Some nursing students panic because they might have repeated a year. Most of our students have serious social problems that affect their academic performance.’ (001-B-21-03)

The ability to effectively integrate knowledge into practice is key to achieving successful outcomes and patient safety among nursing students, for them to excel in nursing practice.

#### Sub-theme 2.2: Lack of a supportive environment

Participants reported a need for a positive and supportive environment to allow nursing students to learn appropriately and feel supported. The supporting environment involves positive attitudes of professionals, resources and the environment. Therefore, the clinical environment should be prepared before nursing students go for their placements. This was stated as follows:

‘We [*nurse educators*] need to know how to integrate theory into the clinical environment in the wards. If our colleagues are not supportive, that is where we learn about supportive environments where need to take over and mentor them. Otherwise, we will have a problem with the learning of our nursing students, as they will be incompetent on completion.’ (001-B-21-03)

‘They [*nursing students*] might feel not welcomed by the clinical staff, not demonstrating procedures to them, and not supervising them in the wards. Nursing students get anxious when the clinical staff do not support and does not mentor them.’ (003-B-21-02)

‘Our students need a lot of support, especially in the facilities, otherwise, they will not be competent nurses on completion.’ (002-C-21-FG20)

A positive, supportive environment is important to support nursing students for them to succeed academically.

#### Sub-theme 2.3: Chronic medical health challenges

Participants revealed that some nursing students have chronic medical conditions, which challenge them, as they need to be on repeat medication and attend follow-up investigations. This was stated as follows:

‘Health challenges that make the student absent from class for a long period becomes a problem for them as they need to catch up afterwards, and their anxiety levels increase.’ (002-B-21-02)

‘Some nursing students need to attend to their appointments for their health issues.’ (003-B-21-01)

Nursing students experience challenges balancing their academic workload, attending their chronic health appointments and other general issues; hence, anxiety levels escalate. They must be supported for academic performance.

#### Sub-theme 2.4: Social influences

According to nurse educators and counsellors, social influences play a significant role in increasing anxiety because of social challenges they are experiencing. Participants noted that most nursing students had social issues triggering anxiety. This was explained as follows:

‘It is multifactorial, but they do write to say that it is personal. This is what we get when we interview the nursing students, checking where the problem is, that’s when we realise that it is multifactorial. Anxiety among nursing students emanates from, social, and it overwhelms them as they do not have a resilient nature.’ (001-B-21-03)

‘Anxiety among nursing students emanates from, social, and it overwhelms them. At times nursing students end up committing suicide that is emanating from social problems.’ (003-B-21-01)

Understanding the influences of anxiety-related conditions is essential as it can be handled better when there is knowledge of the cause. Nursing students can be referred appropriately to alleviate the strain on academic activities.

### Theme 3: Responses of anxiety-related conditions among nursing students

Participants emphasised that nursing students who experience anxiety-related conditions exhibit a wide range of symptoms when they are anxious. These symptoms encompass physical and emotional responses to anxiety-related conditions.

#### Sub-theme 3.1: Physical symptoms

Physical responses to stress are sweaty palms, breathlessness, racing heartbeat, hysteria, unable to talk, crying and fatigue. This was articulated as follows:

‘Some nursing students come to the counselling department when they are panicking with sweaty palms, breathlessness and unable to cope.’ (001-B-21-03)

‘Students will panic, stammer, shiver, some even cry, even unable to answer due to anxiety.’ (001-C-21-FG06)

Nurse educators need to recognise these signs of anxiety to provide prompt support and some resources to help nursing students manage their anxiety and stress effectively.

#### Sub-theme 3.2: Emotional symptoms

Emotional responses in anxiety-related conditions displayed by nursing students are irritability, fear and uneasiness. In addition, nursing students would report suicidal thoughts. This was expressed by counsellors as follows:

‘Our nursing students are struggling, anxiety overwhelms them, it becomes too much for them. They feel so hopeless. hallucinating with suicidal thoughts.’ (001-B-21-03)

‘Some [*nursing students*] will come to my office before test crying, saying that they have a mental block, these students also report being afraid, irritable, and reporting to be scared.’ (003-B-21-01)

Recognising emotional symptoms is crucial in providing timely and targeted support to efficiently help nursing students manage and alleviate their anxiety-related challenges.

### Theme 4: Support of nursing students with anxiety-related conditions

The participants shared the support given when nursing students become anxious during teaching and learning. They use some constructive methods and some advice given to support them. Three sub-themes emerged from this theme: practical advice given to nursing students to manage anxiety-related conditions, referring them to relevant professionals and encouraging them to use available support.

#### Sub-theme 4.1: Practical advice given to nursing students with anxiety-related conditions

Participants shared a variety of advice aimed at alleviating anxiety among nursing students. Their insights highlighted practical strategies to help nursing students effectively manage anxiety during their studies. The advice was to use constructive methods when anxious, including structured study timetable, encouraging deep breathing exercises, carrying a pocketbook to write down difficult questions, time management and reading questions slowly to ensure understanding before tests and exams. This was expressed as follows:

‘I always encourage the nursing students to do deep breathing exercises, support from peers before the skill as it helps a lot reduce anxiety.’ (001-C-21-FG06)

‘I will also tell nursing students that during tests and exams, to read the question slowly and ensure that they understand before they answer.’ (001-C-21-FG07)

‘I will advise the student to carry a pocketbook so that they ask and know in each facility how problems are solved, helping them understand the prevalent question.’ (002-C-21-FG18)

Managing anxiety-related conditions in nursing students is crucial for nurse educators and counsellors, which involves enforcing practical, constructive methods to enhance academic success. The practical advice promotes relaxation and boosts self-esteem.

#### Sub-theme 4.2: Referral to relevant professionals

Using a referral system is vital for the continuous support of nursing students during their training. Nurse educators refer nursing students to the counselling department who become anxious in class or with poor academic performance. Such referrals are directed towards various resources, including fellow nurse educators, pastors, the counselling department and peer support networks. The counsellors conduct their basic psychosocial assessment and refer nursing students to psychologists to further manage anxiety-related conditions. This was narrated as follows:

‘Students who have severe anxiety are referred to psychologists for further management depending on the individual’s situation.’ (002-B-21-04)

‘One-to-one counselling does help the students to cope as it allays their anxiety with nurse educators and nursing students can be referred to preferred nurse educators for consultation.’ (002-C-21-FG13)

The referrals underscore the importance of a multifaceted approach to nursing students’ well-being, emphasising emotional support that is essential to nurturing the next generation of the nursing profession.

#### Sub-theme 4.3: Availability of support system

Nurse educators mentioned they mentor them and provide clinical accompaniment to achieve their work-integrated learning requirements. Nursing students are encouraged to use the available services in the NEIs, like consulting with nurse educators where they can be able to explore their feelings. This was expressed as follows:

‘It’s mentorship, students are accompanied. These are the two mechanisms, for support. However, I can say accompaniment can be the main contributor because we have a ratio where we are expected to do one hour a month of accompaniment. We can also give wards objectives, so that when the students go to the wards the sisters should know that this student is level two, also to allocate students to those colleagues that are interested in students.’ (003-B-21-02)

‘[*Nursing students*] are encouraged to use support services which are available in NEI, usually when I go to class for allocated block period, is then that I realise that students need assistance.’ (002-B-21-04)

‘As soon as the nurse educator picks up anxiety from the student, she should reassure the student, and allow the student to verbalise their feeling. Do not judge or make comparisons with others. Allow the student to consult individually.’ (001-C-21-FG09)

Nurse educators encourage nursing students to openly share their feelings, particularly when signs of anxiety are evident. Nursing students are empowered to take charge of their well-being and make use of the support provided in NEIs. Severe anxiety leads to suicidal thoughts and absences from academic commitments.

## Discussion

The main objective of this article was to evaluate and describe the knowledge of counsellors and nurse educators’ impact of anxiety-related conditions on nursing students’ academic excellence. Four themes emerged from the data: understanding of anxiety-related conditions, influences of anxiety-related conditions, responses of anxiety-related conditions among nursing students and support of nursing students with anxiety-related conditions.

### Understanding of anxiety-related conditions

Participants highlighted the impact of anxiety-related conditions on nursing students as a range of emotions that impede their normal functioning and coping abilities. Participants noted that anxiety-related conditions can overwhelm nursing students, leading to a narrowed focus on short-term solutions and even experiencing panic responses. These conditions severely hinder nursing students’ coping mechanisms and distort their perception, making it difficult for them to manage their academic workload effectively. Keltner and Steele (2019:311) highlight that anxious individuals often struggle to control their worry, which can become a coping mechanism to prevent negative outcomes. The continuous state of worry can hinder problem-solving skills and inhibit the learning process, especially during severe anxiety attacks (Halter 2018:272). The consequences of anxiety-related conditions among nursing students are significant, often leading to absences from academic commitment and poor academic performance (Magobolo & Dube 2019:2). Severity of anxiety feelings in university students leads to suicidal thoughts and a high risk of suicide (McLafferty et al. 2021:4). Understanding anxiety-related conditions is crucial for counsellors and nurse educators, as it enables them to better manage and support nursing students.

### Influences of anxiety-related conditions

Anxiety-related conditions among nursing students are multifaceted, encompassing social, environmental, academic, financial and chronic medical health factors. Participants in the study highlighted that nursing students come from diverse sociocultural backgrounds, which can negatively impact their educational journey and heighten anxiety levels. The recognition of social challenges among nursing students holds significant implications for healthcare service planning and delivery, emphasising the importance of addressing mental health issues within this population (Bantjes et al. 2019:9). Work-integrated learning presents additional stressors for nursing students, including encountering unfamiliar diseases, adapting to new clinical settings, facing limited nursing resources and struggling with success (Laishram & Managiyarkkaraski 2019:1). Vasugi and Hassan (2019:1) highlighted that pressure to excel in an uncertain future exacerbates these challenges. Financial constraints stemming from social issues can further aggravate anxiety levels among nursing students, impacting their academic success (Mofatteh 2020:43). Moreover, chronic medical health conditions can serve as contributory factors to anxiety, further delaying academic achievements (Misgan & Belete 2021:2). Understanding the triggers of anxiety-related conditions is paramount for counsellors and nurse educators. Focussing on the causes, rather than just the symptoms, is imperative for tailored and effective management.

### Responses to anxiety-related conditions

Participants noted that the responses to anxiety-related conditions among nursing students encompass both physical and emotional symptoms. Physically, nursing students experience anxiety, which may exhibit symptoms such as sweaty hands, fatigue, breathlessness and headaches during the attacks. Emotionally, they display irritability, fear, uneasiness, worry and even thoughts of suicide. These observations align with findings in the existing literature, which highlight that individuals facing anxiety often experience a combination of physical and emotional symptoms. Middleton (2020:285) emphasises the physical manifestations of anxiety, including breathlessness, palpitations, headaches and emotional symptoms such as irritability, fear, worry and confusion. Adwas, Jbireal and Azab (2019:2) further assert that anxiety-related conditions are characterised by signs and symptoms such as fear, hopelessness and worry, often accompanied by physical manifestations such as chest pain, dizziness and shortness of breath. Most cultures interpret these behaviours in different ways according to their knowledge and understanding of mental illnesses based on complexity and cultural relativity, which is managed according to the belief system (Bhugra, Watson & Wijesuriya 2021:1). The effects of anxiety can result in chaotic behaviour, both physical and emotional. However, once these symptoms are understood, proper support can be offered to ease them and enable nursing students to function better.

### Support of nursing students with anxiety-related conditions

Participants in the study suggested a multifaceted approach in support of nursing students with anxiety-related conditions. Some key strategies mentioned include stress management techniques such as breathing exercises, singing and walking. These techniques can help alleviate anxiety symptoms and manage stress more effectively. It was emphasised that nursing students should be referred to mental health professionals for effective anxiety management when needed. Seeking professional help is crucial when mental health concerns significantly interfere with academic success (Amen 2020:97). Zhou et al. (2022:1) emphasise the importance of having a dependable social network and perceived support for individuals to rely on during times of need. Nursing students receive support from various sources, including nurse educators, families and friends (Langtree et al. 2018:94). Untreated poor mental health can cause distress among students and negatively influence their quality of life and academic performance (Mofatteh 2020:2). Implementing these strategies, NEIs can create a supportive environment that helps nursing students effectively manage anxiety-related conditions and succeed academically. It is crucial to provide a holistic support system that addresses the physical, emotional and social aspects of anxiety to promote well-being and academic success.

### Limitations

Data were collected during level 3 COVID-19 pandemic restrictions. Virtual interviews created challenges, such as connectivity arising from poor signals and the need for clarification during data collection. The researcher could not observe non-verbal communication. The findings cannot be generalised because the study was qualitative.

### Recommendations

Integrate mental healthcare awareness modules into the nursing curriculum focussing on anxiety. Understand the impact of mental health and strategies for coping and establishing peer support. Programmes like these can play a role in reducing isolation and stigma associated with anxiety-related conditions. Ensuring that counsellors receive training will make their services readily available and accessible in the NEIs. Foster collaboration between NEIs and mental healthcare professionals, which might provide expertise to support nursing students. Nursing education institutions can create an environment that is conducive to academic success and well-being by making support information readily available to nursing students.

## Conclusion

The study findings underscore the significant negative impact of anxiety-related conditions on nursing students within NEIs. These conditions manifest in emotional and physical symptoms, detrimentally affecting academic performance. Nursing education institutions are faced with challenges because of nursing students experiencing anxiety-related conditions that exert a burden on academic success. Prioritising the mental health and well-being of nursing students is crucial for ensuring they are both mentally and academically successful upon completion.
